# Type I Interferon Modulates the Function of Ly6C High-Expressing Naïve CD8^+^ T Cells to Promote an Antitumor Response

**DOI:** 10.3390/vaccines13030246

**Published:** 2025-02-27

**Authors:** Hsin-Fang Tu, Julia Tao, Ming-Hung Hu, Darrell Fan, Ya-Chea Tsai, Tzyy-Choou Wu, Chien-Fu Hung

**Affiliations:** 1 Department of Pathology, Johns Hopkins University School of Medicine, Baltimore, MD 21205, USA; 2 Department of Surgery, Yale School of Medicine, Yale University, New Haven, CT 06510, USA; 3 Department of Oncology, Johns Hopkins University School of Medicine, Baltimore, MD 21205, USA; 4 Department of Obstetrics and Gynecology, Johns Hopkins University School of Medicine, Baltimore, MD 21205, USA; 5 Molecular Microbiology and Immunology, Johns Hopkins University School of Medicine, Baltimore, MD 21205, USA

**Keywords:** type I interferon, Ly6C^hi^ CD8 T cells, immune modulation, cancer immunotherapy

## Abstract

**Background:** Ly6C expression in naïve CD8+ T cells plays a crucial role in enhancing their effector activity, suggesting potential implications for cancer immunotherapy. This study investigates the functional impact of Ly6C expression on CD8^+^ T cells and explores albumin-conjugated IFNβ (Alb-IFNβ) as a strategy to modulate Ly6C expression and improve cancer vaccine efficacy. **Methods:** We analyzed the functional differences between Ly6C high-expressing (Ly6C^hi^) and Ly6C low-expressing (Ly6C^lo^) naïve CD8^+^ T cells in tumor suppression. To assess the role of type I interferon signaling, we administered Alb-IFNβ in C57BL/6J and IFNAR^−/−^ mice and measured Ly6C expression in CD8^+^ T cells. The therapeutic potential of Alb-IFNβ was further evaluated in combination with a vaccinia virus encoding the HPV-16 E7 antigen (CRT-E7 vaccine) in a syngeneic TC-1 tumor model, assessing tumor growth, survival, and antigen-specific CD8^+^ T cell responses. **Results:** Naïve CD8^+^ T cells with elevated Ly6C expression exhibited enhanced tumor-suppressive capacity and required lower activation thresholds for effector function. Alb-IFNβ treatment selectively increased Ly6Chi naïve CD8^+^ T cells in C57BL/6J mice but not in IFNAR^−/−^ mice, confirming type I interferon’s role in Ly6C regulation. Combining Alb-IFNβ pretreatment with the CRT-E7 vaccine significantly enhanced antigen-specific CD8^+^ T cell immunity, reducing tumor growth and prolonging survival in TC-1 tumor-bearing mice. **Conclusions:** Our findings suggest that Alb-IFNβ may enhance the antitumor activity of naïve CD8^+^ T cells by modulating Ly6C expression. Alb-IFNβ could potentially improve the efficacy of HPV vaccinia-based cancer vaccines, warranting further investigation as an adjuvant strategy in cancer immunotherapy.

## 1. Introduction

Immune diversification is crucial in enabling the host to fight against various pathogens and halt cancer progression, with CD8^+^ T cells playing a critical role in this process. The heterogeneity and diverse functionality of effector and memory CD8^+^ T cells are well established. However, the variability among naïve CD8^+^ T cells before encountering a specific antigen has been underappreciated [1,2]. Recent studies have revealed heterogeneity within CD8^+^ T cell populations, contributing to immune diversification to assist the host against a wide array of pathogens. It has been reported that naïve CD8^+^ T cells are equipped with different functional capabilities based on various surface proteins such as CD5, Ly6C, CD183, and GM1 [3,4,5]. These variations are observed within individual T cell receptor (TCR) specificities and in cell activation and homeostasis.

Ly6C is a glycosylphosphatidylinositol (GPI)-anchored cell surface protein expressed on various immune cells, including T cells, macrophages, and dendritic cells [6,7]. It is commonly used as a marker to differentiate between classical and non-classical monocytes/macrophages and has been reported as an accessory molecule in the cytolytic function of cytotoxic T lymphocytes [8]. Additionally, its expression on T cells is positively correlated with better T cell proliferation and IL-2 production upon activation [9]. Recent studies on Ly6C-expressing naïve CD8^+^ T cell subsets have revealed some fascinating findings, highlighting their crucial roles in antitumor and antiviral immunity. These cells exhibit a transcriptome more similar to activated effectors than Ly6C^lo^ naïve CD8^+^ T cells. As a result, they produce stronger immune responses when activated. The underlying mechanism behind this is driven by type I interferon (IFN) signaling, which leads to distinct effector responses [5,10]. Moreover, the administration of Poly(I:C) to induce type I IFN signaling could sensitize naïve CD8^+^ T cells to immediate effector functions on exposure to a cognate ligand [11]. These results reveal multiple functional features of Ly6C^hi^ naïve CD8^+^ T cells. It is worth investigating whether this specific immune subset can contribute to tumor control and improve the effectiveness of cancer immunotherapy.

Type I IFNs are crucial cytokines for antiviral immunity, bridging innate and adaptive immune responses [12]. One of the challenges to using cytokine treatment in clinics, such as IFNβ, is its short serum half-life in humans [13,14]. Recent advancements include albumin conjugation, a technique that could substantially extend the lifespan and enhance the efficacy of protein-based treatments [15,16]. Using the albumin conjugation technique, our group successfully generated several immunomodulatory molecules for both vaccination and anticancer purposes in the past, including albumin (Alb)-GM-CSF, Alb-IL2, and Alb-IFNβ [17,18,19]. To overcome the pertinent challenge of using IFNβ in treatment, we genetically fused albumin with IFNβ to create Alb-IFNβ. This extended its half-life and improved cross-presentation by antigen-presenting cells (APCs), leading to stronger antigen-specific T cell and B cell responses. Additionally, the ability of Alb-IFNβ to serve as an adjuvant was tested when combined with HPV vaccines [19].

In this study, we evaluated the differences in the functional potential between Ly6C high-expressing (Ly6C^hi^) and Ly6C low-expressing (Ly6C^lo^) naïve CD8^+^ T cells to control tumor growth. We demonstrated that type I IFN signaling, such as IFNβ, is important in maintaining Ly6C expression in CD8^+^ T cells, thus improving their activation and effector functions. Subsequently, we highlighted the therapeutic potential of Alb-IFNβ in preconditioning CD8^+^ T cell functions in vivo and in vitro. The administration of Alb-IFNβ in combination with our vaccinia virus encoding the HPV-16 E7 antigen (HPV vaccinia-based vaccine) enhanced antigen-specific CD8^+^ T cell immunity in a syngeneic TC-1 tumor model. These results suggest that Alb-IFNβ could serve as a potent adjuvant for the HPV vaccinia-based vaccine in the treatment of HPV antigen-expressing tumors. Overall, our data suggest that leveraging Alb-IFNβ to precondition naïve T cells may have significant potential in cancer immunotherapy for the control of infectious disease and cancer.

## 2. Material and Methods

### 2.1. Reagents and Antibodies

Alb-IFNβ was generated from pcDNA3-Alb-IFNβ utilizing the previously described method [19]. The pcDNA3-Alb-IFNβ construct was created by amplifying mouse IFNβ from the pUNO1-mIFNB1 plasmid and cloning it into pcDNA3-Alb, with verification by DNA sequencing. The expression of Alb-IFNβ proteins was achieved in Expi293F cells using a kit from Thermo Fisher Scientific (Waltham, MA, USA), followed by purification with a HiTrap albumin column from GE Healthcare Life Sciences.

The antibodies and reagents used in the flow cytometry analysis are shown in Table 1.

### 2.2. Ethics Approval

All animals were housed and handled in the animal facility of the Johns Hopkins Medical Institution under specific pathogen-free conditions. All procedures were performed according to the approved protocols by the Johns Hopkins Medical Institutions Animal Care and Use Committee and the National Institutes of Health.

### 2.3. Mice

C57BL/6 female mice aged 6–10 weeks were purchased from Taconic Biosciences (Germantown, NY, USA). OT-1 and B6(Cg)-Ifnar1tm1.2Ees/J mice were purchased from Jackson Laboratories (Farmington, CT, USA). All mice were maintained at the Johns Hopkins University School of Medicine (Baltimore, MD, USA) animal facility under specific pathogen-free conditions.

### 2.4. Cell Culture

Mouse melanoma B16ova cells were acquired as a generous gift from Dr. Charles Drake and maintained in complete DMEM media supplemented with 10% FBS, 1% L-glutamine, 100 U/mL penicillin, 100 mg/mL streptomycin, 2 mM sodium pyruvate, and 2 mM non-essential amino acid. TC-1 tumor cells were generated and maintained as previously described [20].

### 2.5. In Vitro Stimulation of Naïve T Cell

Lymphocytes were isolated from pooled spleens and lymph nodes of C57BL/6 mice. Red blood cells (RBCs) were lysed twice using an excess of RBC lysis buffer (Cell Signaling Technology, Danvers, MA, USA), followed by extensive washing with 0.5% BSA/PBS (FACS buffer). Naive CD8^+^ T cells were then isolated using the EasySep™ Mouse Naive CD8^+^ T Cell Isolation Kit (StemCell Technologies, Vancouver, BC, Canada), and the isolation efficiency was confirmed by flow cytometry (Figure S1). To assess the activation and effector functions of naive CD8^+^ T cells under IFN-β regulation, the cells were preconditioned with 50 ng/mL IFNβ for 24 h before being stimulated with anti-CD3 (1.25 μg/mL) and anti-CD28 (0.25 μg/mL) antibodies for an additional 24 h. Subsequently, the cells were harvested and analyzed by flow cytometry.

### 2.6. Type I IFN-Mediated Immunomodulation of Ly6C Expression in CD8⁺ T Cells

For in vitro experiments, naive CD8⁺ T cells were isolated from C57BL/6J and IFNAR^−/−^ mice. The cells were then incubated with 50 ng/mL of type I interferons (IFNα or IFNβ1) for 24 h. Ly6C expression in CD8⁺ T cells was assessed by flow cytometry. For in vivo analysis, C57BL/6 mice received a single dose of IFNβ (30 μg). Peripheral blood mononuclear cells (PBMCs) were collected on days 2, 7, 14, 21, and 28 and analyzed by flow cytometry to evaluate Ly6C expression.

### 2.7. Flow Cytometry and Cell Sorting

Blood samples were collected from mice via facial vein lancet puncture into EDTA-coated tubes. Lymphocytes from lymph nodes or spleens were processed into single-cell suspensions. Red blood cells (RBCs) were lysed using RBC lysis buffer (Cell Signaling Technology, Danvers, MA, USA) twice, followed by extensive washing in 0.5% BSA/PBS (FACS buffer). Compensation for each experiment was set using single staining controls of UltraComp eBeads (Thermo Fisher Scientific, Waltham, MA, USA). Fluorescence minus one (FMO) and isotype controls were employed for the accurate gating and assessment of non-specific binding. Zombie Aqua dye (BioLegend, San Diego, CA, USA) was applied for dead cell exclusion as per the manufacturer’s protocol before antibody staining. Prior to antibody staining, Fc Block was applied to minimize non-specific binding. Optimal concentrations for antibody and tetramer use were established through titration. Antibody staining was conducted for a minimum of 30 min at 4 degrees Celsius. Data acquisition was performed using a 13-color Beckman Coulter CytoFLEX S flow cytometer, with analysis carried out in FlowJo 10.4 software (FlowJo LLC). The BD FACSAria™ Fusion sorter at the Johns Hopkins School of Medicine CRB HP Flow Core facility was utilized for cell sorting of Ly6C^hi^ or Ly6C^lo^ naïve CD8^+^ T cells from OT-1 mice. The naïve CD8^+^ T cells were resuspended in 10% RPMI/FBS and stimulated with OVA257-264 (SIINFEKL, ranging from 0.0625 to 0.5 ng/mL) peptides for 24 h. To assess cytokine production, mouse lymphocytes were incubated with the protein transport inhibitor Brefeldin A (BioLegend, San Diego, CA, USA) for 16 h. Following this, cells were processed as outlined in the flow cytometry protocol. After extracellular staining, cells were permeabilized using the eBioscience Foxp3/Transcription Factor Staining Buffer Set (Thermo Fisher Scientific, Waltham, MA, USA) and stained for intracellular cytokines.

### 2.8. Adoptive Cell Transfer

Lymphocytes were isolated from the spleen and lymph nodes of OT-1 mice and processed into single-cell suspensions. Red blood cells (RBCs) were lysed using RBC lysis buffer (Cell Signaling Technology, Danvers, MA, USA) twice, followed by thorough washing in 0.5% BSA/PBS (FACS buffer). The cells were stained with fluorescently conjugated antibodies targeting CD3, CD8, CD44, CD62L, and Ly6C, washed, and resuspended in staining buffer. Naïve CD8^+^ T cells, both Ly6C^hi^ and Ly6C^lo^, were sorted using flow cytometry. For adoptive transfer experiments, 2 × 10^6^ T cells were administered through retro-orbital injection in 100 μL of PBS.

### 2.9. Tumor Experiments

In the tumor experiments, 2 × 10^5^ B16-ova cells and 1 × 10^5^ TC-1 cells were subcutaneously inoculated on the flank of C57BL/6 mice. Tumor size was monitored with a digital caliper and calculated using the formula length × width^2^ × 0.5. The experiments concluded when tumors exceeded 2 cm in diameter or if mice exhibited weight loss, adhering to the approved animal protocols. Tumors and draining lymph nodes were collected for flow cytometry. Draining lymph nodes were processed into single-cell suspensions, underwent RBC lysis, were washed, and were finally resuspended in FACS buffer for analysis. For leukocyte analysis within tumors, tissues were placed in FACS buffer containing magnesium and calcium, minced into ~2 mm pieces, and digested with Collagenase I, Collagenase IV, and DNase I for 20 min at 37 °C. The reaction was quenched with 10% RPMI/FBS, and samples were then centrifuged and passed through a 70 μm cell strainer. Tumor samples were further purified using a Ficoll gradient. Cells were resuspended in 10% RPMI/FBS, layered onto Ficoll-Paque Plus (GE Healthcare Life Sciences, Marlborough, MA, USA), and centrifuged at 400× *g* at 25 °C for 30 min. The lymphocyte layer was collected, washed in PBS, and centrifuged again. After counting, cells were prepared for flow cytometry.

### 2.10. Vaccinia and Vaccination

The method for generating the vaccinia CRT-E7 vaccine has been previously described [21]. Viral stocks were stored at −80 °C. Prior to use, the virus was thawed and treated with a 1/10 volume of trypsin/EDTA in a 37 °C water bath for 30 min and then diluted with minimal essential medium (MEM) containing 2.5% fetal bovine serum to a final concentration of 1 × 10^8^ plaque-forming units (PFUs)/mL. Mice were immunized by intraperitoneal (i.p.) injection with a single dose of the diluted vaccine (0.1 mL).

### 2.11. Statistical Analysis

All data are expressed as means ± standard error of the mean (S.E.M). The statistical significance was determined by a one-way ANOVA with the Tukey–Kramer multiple comparison or Student’s *t*-test using Prism 9 software (GraphPad, CA, USA). In all circumstances, *p*-values ≤ 0.05 were considered significant (*, *p* < 0.05; **, *p* < 0.01; ***, *p* < 0.001; ****, *p* < 0.0001).

## 3. Results

### 3.1. Naïve CD8^+^ T Cells with High Ly6C Expression Enhance Effector Functions and Tumor Control

**Figure 1:**
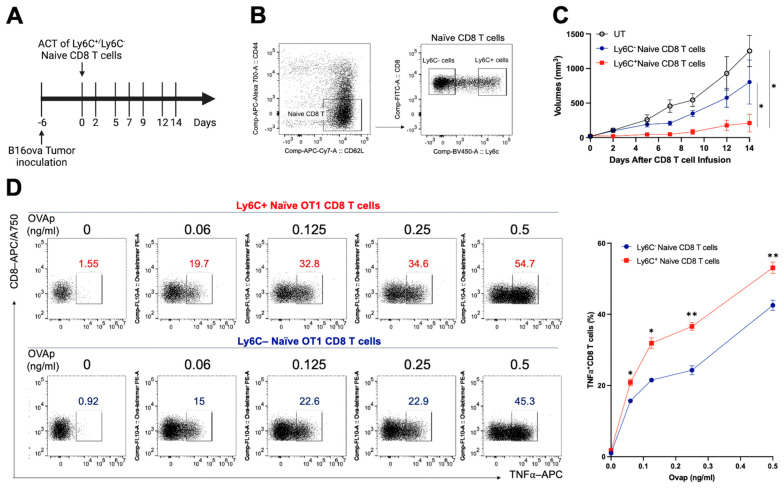
**Adoptive cell transfer of Ly6C^hi^ naïve OT1 CD8 T cells generates superior tumor control capability.** (**A**) C57BL/6 mice were subcutaneously inoculated with B16ova cells. After six days, the tumor-bearing mice received a retro-orbital sinus injection of flow-sorted Ly6C^hi^or Ly6C^lo^ naïve CD8 T cells (5 mice per group) isolated from OT-1 mice. Tumor sizes were monitored every two to three days with a caliper. (**B**) The representative figure shows the gating strategy for Ly6C expression on naïve CD8 T cells used for cell sorting. (**C**) The tumor growth curve following the treatment protocol. (**D**) Ex vivo assessment of T cell effector function in both sorted Ly6C^hi^ or Ly6C^lo^ CD8 T cells by stimulation with varying concentrations of OVA257-264 (SIINFEKL) peptides for 16 h. The frequency of TNFα expression in total CD8 T cells was analyzed by flow cytometry. In all circumstances, *p*-values ≤ 0.05 were considered significant (*, *p* < 0.05; **, *p* < 0.01).

While Ly6C proteins are useful markers for cellular differentiation and subset identification, their physiologic function is less well comprehended. To better understand whether naïve CD8^+^ T cells with high Ly6C expression have improved effector function and tumor control capability, we used cell sorting to isolate Ly6C^hi^ and Ly6C^lo^ naïve CD8^+^ T cells from OT1 mice and used adoptive cell transfer to administer these cells in a B16ova tumor-bearing mouse model (Figure 1A,B). B16ova tumor-bearing mice that received Ly6C^hi^ CD8^+^ T cells displayed substantially delayed tumor growth compared to the untreated group and the group that received adoptive cell transfer of the Ly6C^lo^ CD8^+^ T cells (Figure 1C). Furthermore, a comparison of the functional difference between these two subsets of CD8^+^ T cells by stimulating T cell activation in vitro with different concentrations of ovalbumin (OVA) peptide (257–264) indicated that Ly6C^hi^ CD8^+^ T cells produced more TNF⍺ expression upon peptide stimulation, suggesting an enhanced CD8 T cell effector function compared to Ly6C^lo^ CD8^+^ T cells (Figure 1D). Notably, a low dose (0.06 ng/mL) of the peptide was sufficient to trigger a comparable level of TNF⍺ secretion in Ly6C^hi^ naïve CD8^+^ T cells as a higher dose (0.125 ng/mL) did in Ly6C^lo^ naïve CD8^+^ T cells. It revealed that Ly6C^hi^ CD8^+^ T cells possess a lower activation threshold, reducing the antigen quantity necessary for their effector function. These observations collectively imply that Ly6C^hi^ CD8^+^ T cells are more effective in tumor growth suppression compared to Ly6C^lo^ naive CD8^+^ T cells, partially due to enhanced effector functions.

### 3.2. Type I IFN Signaling Modulates Naïve CD8 T Cell to Express Ly6C^hi^ Features

**Figure 2:**
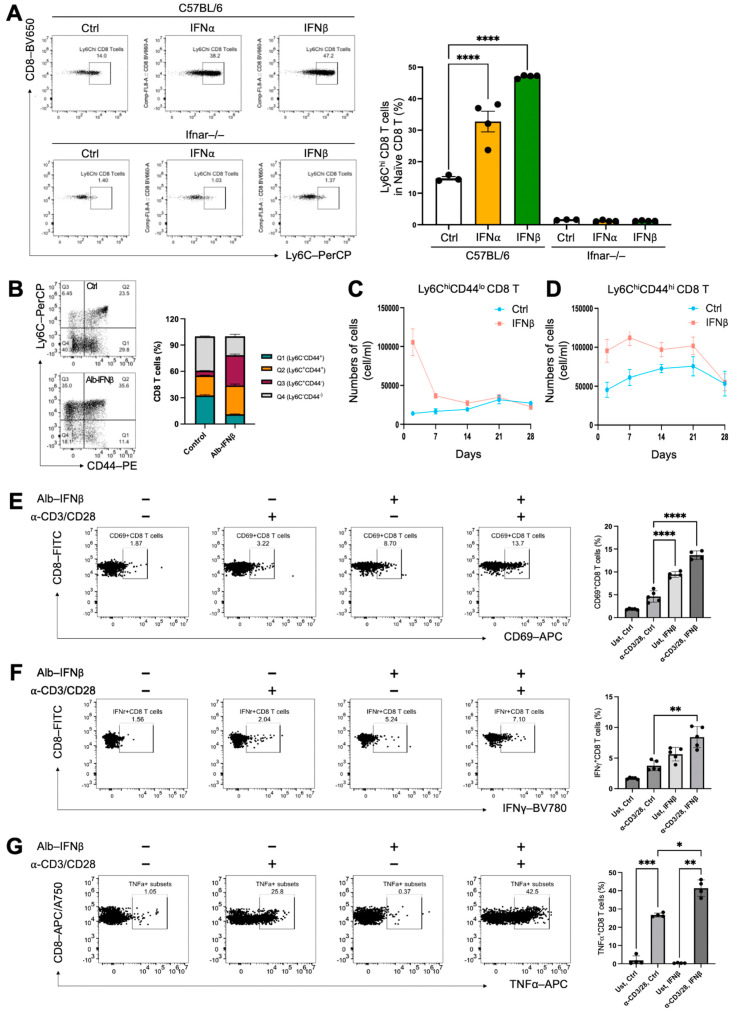
Type I interferon preconditioning upregulates Ly6C expression on naïve CD8 T cells, enhancing effector functions. (**A**) Magnetic beads were used to isolate the naïve CD8 T cells from 6-week-old C57BL/6 (n = 3) or 6-week-old B6(Cg)-Ifnar1tm1.2Ees/J (n = 3) mice. Cells were incubated with 50 ng/mL of type I interferons IFNα or IFNβ1 for 24 h. The figure depicts the frequencies of Ly6C^hi^ subsets in CD3^+^CD8^+^ T cells for the indicated treatment groups. (**B**–**D**) To evaluate the effect of type I interferon on Ly6C expression in CD8 T cells, C57BL/6 mice (n = 5 per group) received a single dose of IFNβ (30 μg) or PBS control. PBMCs were collected on days 2, 7, 14, 21, and 28 and analyzed by flow cytometry. Representative gating of CD8 T cell subsets based on CD44 and Ly6C expression is shown for day 2 (**B**). The line graph illustrates the temporal dynamics of Ly6C⁺CD44⁻ (**C**) and Ly6C⁺CD44⁺ (**D**) CD8 T cell populations. (**E**–**G**) To evaluate the functional differences in Alb-IFNβ preconditioned CD8 T cells, naïve CD8+ T cells from 6-week-old C57BL/6 mice (n = 4) were isolated using magnetic beads and treated with 50 ng/mL Alb-IFNβ for 24 h, followed by stimulation with CD3 and CD28 antibodies for another 24 h. (**D**–**F**) The frequencies of CD69+ (**E**), IFNγ+ (**F**), and TNFα+ (**G**) in total CD8 T cells in C57BL/6 mice were examined across the specified treatment groups. In all circumstances, *p*-values ≤ 0.05 were considered significant (*, *p* < 0.05; **, *p* < 0.01; ***, *p* < 0.001; ****, *p* < 0.0001).

Previous studies have indicated that type I IFN signaling is crucial in the modulation of Ly6C expression in naive mouse CD4^+^ and CD8^+^ T cells [5,10,22]. To validate the importance of type I IFNs in inducing Ly6C expression in vitro and in vivo, we first conducted an in vitro experiment where we isolated naive CD8^+^ T cells from naive C57BL/6J and IFNα receptor-deficient B6/J (IFNAR^−/−^) mice. Subsequently, the naive CD8^+^ T cells were treated with two type I IFNs (IFNα and IFNβ1) for 24 h. The results revealed a significant increase in the frequencies of Ly6C^hi^ naive CD8^+^ T cells in IFNα- and IFNβ1-treated cells from C57BL/6J mice, but not in IFNAR^−/−^ mice (Figure 2A). This suggests that the modulation of Ly6C expression by IFNα and IFNβ1 is mediated through type I IFN signaling pathways. To verify the in vivo immunomodulation of type I IFN on naïve CD8 T cells, Ly6C expression levels were assessed in the peripheral blood of C57BL/6J mice two day after Alb-IFNβ treatment. The results similarly showed significantly enhanced frequencies of Ly6C^hi^ CD8^+^ T cells in the Alb-IFNβ-treated group (Figure 2B). Furthermore, as CD44 is a marker of T cell activation and an indicator of long-lived memory T cells or antigen-experienced cells, we investigated whether Alb-IFNβ-induced Ly6C^hi^ CD8^+^ T cells featured activated and memory phenotypes. The results suggested that Ly6C expression was upregulated after Alb-IFNβ treatment regardless of the CD44 expression levels. We were also curious about the longevity of these Alb-IFN induced cells in the peripheral blood. Thus, we collected blood samples from facial veins and traced both Ly6C^hi^CD44^lo^ and Ly6C^hi^CD44^hi^ CD8^+^ T cells from the vehicle control and Alb-IFNβ-treated mice once a week for a total of four weeks. We found that the numbers of Ly6C^hi^CD44^lo^ CD8^+^ T cells continuously decreased following IFNβ induction (Figure 2C). Surprisingly, the Ly6C^hi^CD44^hi^ population was much more stable and was able to last for at least 3 weeks (Figure 2D). These findings suggest an immunomodulatory effect of type I IFN (Alb-IFNβ) on inducing Ly6C expression in naïve CD8^+^ T cells.

### 3.3. IFNβ-Preconditioned Naïve CD8^+^ T Cells Enhance Activation and Effector Functions

To investigate the functional variances of naïve CD8^+^ T cells with Alb-IFNβ treatment, we isolated naïve CD8^+^ T cells from C57BL/6J mice and preconditioned them in vitro with Alb-IFNβ for one day before stimulating them with anti-CD3 and anti-CD28 antibodies for T cell activation. The results demonstrated that Alb-IFNβ preconditioned CD8^+^ T cells were equipped with enhanced CD8 T cell activation (Figure 2E) and improved effector functions, including IFNγ (Figure 2F) and TNFα (Figure 2G) production. Moreover, we noticed that Alb-IFNβ preconditioned CD8^+^ T cells showed a partially activated phenotype characterized by the upregulation of the surface activation marker CD69, even in the absence of T cell stimulants (Figure 2E). These findings demonstrate that preconditioning naïve CD8+ T cells with Alb-IFNβ can enhance effector functions and potentially reduce the T cell activation threshold.

### 3.4. Vaccination with HPV16 Vaccinia CRT-E7 and Alb-IFNβ Increases Tumor-Specific CD8^+^ T Cell Infiltration and Delays Tumor Progression

**Figure 3:**
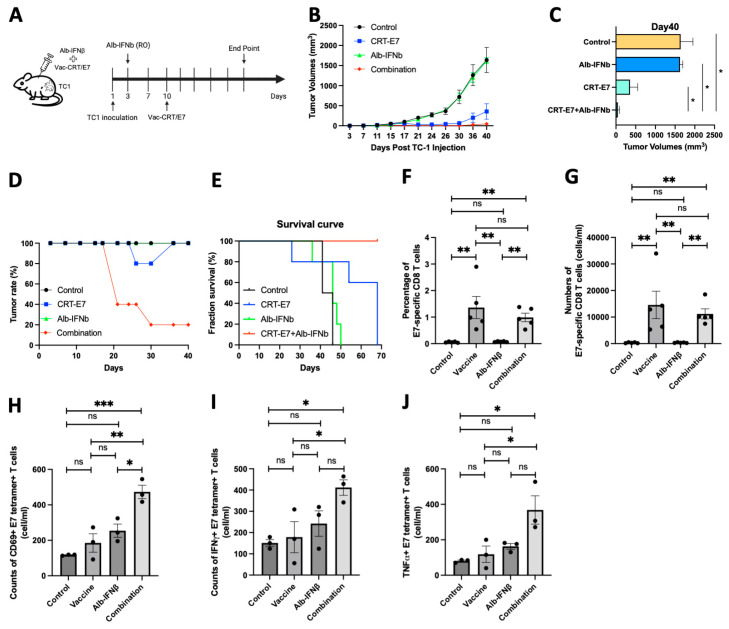
Preconditioning of Alb-IFNβ prior to vaccination with vaccinia-CRT/E7 improves tumor control capability in TC1 tumor-bearing mice. (**A**) C57BL/6 mice were subcutaneously inoculated with TC1 cells. Two days later, mice were injected through the retro-orbital sinus with 30 ug of Alb-IFNb. Seven days after Alb-IFNb treatment, mice were subcutaneously injected with the HPV vaccinia vaccine encoding HPV-16 E7 antigen (n = 5 per group). (**B**) Tumor growth curve following the described treatment protocol. (**C**) Tumor volumes on day 40. (**D**) Tumor rate in the four groups. (**E**) Survival rate in the four groups. (**F**,**G**) The frequencies (**F**) and numbers (**G**) of E7-specific CD8 T cells within the total CD8 T cell population from the peripheral blood one week after Vac-CRT/E7 treatment. (**H**–**J**) The numbers of CD69+ (**H**), IFNγ+ (**I**), and TNFα+ E7-specific CD8 T cells (**J**) in peripheral blood one week after Vac-CRT/E7 treatment. In all circumstances, *p*-values ≤ 0.05 were considered significant (*, *p* < 0.05; **, *p* < 0.01; ***, *p* < 0.001; ns, no significance).

Since the observation that the preconditioning of naïve CD8^+^ T cells can substantially modulate their effector functions once activated, we aimed to understand whether Alb-IFNβ could enhance the efficacy of cancer vaccines. We employed a HPV16 E7-expressing TC1 tumor model and pretreated the mice with Alb-IFNβ before vaccinating them with HPV16 vaccinia CRT-E7 [21] to simulate T cell preconditioning (Figure 3A). The results demonstrated that vaccination combined with Alb-IFNβ significantly delayed tumor growth compared to no treatment, Alb-IFNβ-only, or vaccinia CRT-E7-only groups (Figure 3B,C). Surprisingly, four out of five mice in the combined treatment group were tumor-free (Figure 3D). Additionally, combining Alb-IFNβ with vaccinia CRT-E7 improved overall survival in the mice with tumors (Figure 3E). To assess the E7-specific CD8^+^ T cells induced by the treatment, we conducted E7 tetramer staining on the peripheral blood from different groups of mice. We observed that both vaccinia alone and its combination with Alb-IFNβ could induce high levels of E7-specific CD8^+^ T cells, with no significant differences between them (Figure 3F,G). Further analysis of the activation status by CD69 (Figure 3H) and effector status by IFNγ (Figure 3I) and TNFα (Figure 3J) expression in the E7-specific CD8^+^ T cells revealed higher levels of activated and effector function T cells in the combined treatment group. These findings suggest that Alb-IFNβ can act as an immunomodulator to enhance the effector functions of CD8^+^ T cells against cancer progression.

## 4. Discussion

This research investigates how Ly6C^hi^ naïve CD8^+^ T cells contribute to tumor suppression and the potential effectiveness of cancer vaccines. Our findings reveal that Ly6C^hi^ naïve CD8^+^ T cells exhibit superior tumor control capabilities and require a lower threshold for their effector functions. The administration of Alb-IFNβ not only induces a Ly6C^hi^ phenotype in naïve CD8^+^ T cells but also sensitizes the cells to increase the production of TNFα in response to anti-CD3 and anti-CD28 antibody stimulation. A proof-of-concept experiment involving a combined Alb-IFNβ pretreatment with a vaccinia CRT-E7 vaccine demonstrated an enhanced antitumor immune response and delayed tumor progression, suggesting a key role for the immunomodulation of Ly6C^hi^ naive CD8^+^ T cells in this process. Our findings provide a promising strategy to improve cancer immunotherapy, emphasizing that preconditioning with type I IFN allows naïve CD8^+^ T cells to rapidly engage in effector functions in response to lower antigen levels.

The regulation of Ly6C expression through type I IFN signaling in immune subsets was first reported in hematopoietic cells, which was necessary for the generation of Ly6C^hi^ monocytes and contributed to resistance to influenza infection [23]. Another study found that treatment-induced type I IFN expression affected the recruitment of Ly6C^hi^ monocytes and macrophages to the peritoneal cavity, significantly contributing to the suppression of peritoneal carcinomatosis [24]. It has also been identified that Ly6C is upregulated in central memory as well as effector CD8^+^ T cell subsets [25]. Moreover, recent studies indicate a sub-population of naïve CD8^+^ T cells with Ly6C^hi^ features driven by type I IFN that preferentially homes to lymph nodes and displays enhanced effector functions, suggesting the cytokine modulation of naïve CD8^+^ T cell homeostasis [10].

The phenotype of Ly6C^hi^ naïve CD8 T cells has been explored in previous studies. Notably, both Jergović et al. and Ju et al. conducted RNA-seq analyses on Ly6C^hi^ and Ly6C^lo^ naïve CD8 T cells derived from C57BL/6 mice [1,2]. Differential gene expression analysis revealed that Ly6C^hi^ CD8+ Tn cells co-expressed CD5, CXCR3, Eomes, and Sca-1. This observation was also validated at the protein level through flow cytometry analysis. Collectively, these results suggest that Ly6C^hi^ naïve CD8 T cells may share phenotypic and functional characteristics with VM CD8 T cells. Our results bring new implications for therapeutic strategies leveraging type I IFN signaling to precondition naïve CD8^+^ T for cancer immunotherapy.

Type I IFNs have been shown to critically support the antiviral and antitumoral CD8^+^ T cell response. The enhanced effector capabilities of Ly6C^hi^ naïve CD8 T cells have been reported in several studies. For instance, Jergović et al. demonstrated that Ly6C^hi^ naïve CD8 T cells isolated from secondary lymphoid tissues of SPF mice and stimulated with α-CD3/α-CD28 beads, phorbol-myristate acetate (PMA), and ionomycin exhibited a robust and rapid effector response, producing significantly higher levels of GzB, IFNγ, and TNFα compared to Ly6C^lo^ naïve CD8 T cells [2]. Additional research supports these findings, indicating the self-reactivity and enhanced effector attributes of Ly6C^hi^ naïve CD8 T cells in response to viral infection and sepsis [1,3]. The strength of TCR interactions with peptide-bound MHC (pMHC) influences T cell response kinetics and magnitude [4,5,6]. In this study, we further investigated whether TCR interaction strength differs between Ly6C^hi^ and Ly6C^lo^ naïve CD8+ T cells, comparing their functions by assessing TNFα expression with varying OVA peptide concentrations. The results in Figure 1D not only support previous findings but also highlight that Ly6C^hi^ naïve CD8+ T cells require lower activation thresholds to mount effector immune responses effectively.

## 5. Conclusions

In this study, we confirmed the direct role of type I IFN signaling in the preconditioning of naïve CD8^+^ T cells. Our findings underscore the importance of type I IFN immunomodulation in promoting the development of Ly6C^hi^ naive CD8^+^ T cells, which are characterized by their heightened self-reactivity and enhanced effector capabilities. The ability of APCs to recruit T cells across a spectrum of functional avidities significantly impacts immune response efficacy. This recruitment depends not only on the levels of co-stimulatory and inhibitory molecules but also on the efficiency of antigen presentation. We demonstrated a strategy to improve the functional avidity of naïve CD8^+^ T cells via type I IFNs, providing a potential rationale for reacting low-avidity tumor-specific CD8^+^ T cells to improve cancer immunotherapy outcomes.

## Figures and Tables

**Table 1:** **The antibodies and reagents used in flow cytometry analysis.** The antibodies used are shown, as well as their corresponding catalog number and company.

ANTIBODIES	CATALOG	COMPANY
APC/Cyanine7 anti-mouse CD62L Antibody	104427	BioLegend (San Diego, CA, USA)
BD Horizon™ APC-R700 Rat Anti-Mouse CD44	565480	BD Biosciences (Franklin Lakes, NJ, USA)
Brilliant Violet 421™ anti-mouse Ly-6C Antibody	128031	BioLegend (San Diego, CA, USA)
FITC anti-mouse CD8a Antibody	100705	BioLegend (San Diego, CA, USA)
PerCP/Cyanine5.5 anti-mouse Ly-6C Antibody	128011	BioLegend (San Diego, CA, USA)
APC anti-mouse TNF-α Antibody	506307	BioLegend (San Diego, CA, USA)
APC anti-mouse CD69 Antibody	104513	BioLegend (San Diego, CA, USA)
Brilliant Violet 785™ anti-mouse IFN-γ Antibody	505837	BioLegend (San Diego, CA, USA)
APC/Fire™ 750 anti-mouse CD8a Antibody	100766	BioLegend (San Diego, CA, USA)
Zombie AquaTM Fixable Viability Kit	423102	BioLegend (San Diego, CA, USA)
HLA-A*02:01 HPV16 E7 Tetramer-YMLDLQPETT-PE	TB-M048-1	MBL International (Schaumburg, IL, USA)
Brilliant Violet 650™ anti-mouse CD8a Antibody	100742	BioLegend (San Diego, CA, USA)
Brilliant Violet 785™ anti-mouse CD3 Antibody	100231	BioLegend (San Diego, CA, USA)
CD44 Monoclonal Antibody (IM7), PE	12-0441-82	Thermo Fisher Scientific (San Diego, CA, USA)
FITC Anti-CD8 alpha antibody [KT15]	Ab22504	Abcam (Cambridge, United Kingdom)
Brilliant Violet 785™ anti-mouse CD3 Antibody	100231	BioLegend (San Diego, CA, USA)
EasySep™ Mouse Naïve CD8+ T Cell Isolation Kit	19858	StemCell Technologies (Vancouver, BC, Canada)
Ultra-LEAF™ Purified anti-mouse CD3 Antibody	100238	BioLegend (San Diego, CA, USA)
Ultra-LEAF™ Purified anti-mouse CD28 Antibody	102116	BioLegend (San Diego, CA, USA)
Invitrogen™ eBioscience™ Foxp3/Transcription Factor Staining Buffer Set	50-112-8857	Thermo Fisher Scientific (San Diego, CA, USA)
Recombinant Mouse IFN-α (carrier-free)	752804	BioLegend (San Diego, CA, USA)
Recombinant Mouse IFN-β1 (carrier-free)	581304	BioLegend (San Diego, CA, USA)

## Data Availability

All data relevant to this study are included in the article or uploaded as Supplementary Information. Data and materials are available on reasonable request.

## Supplementary Materials

The following supporting information can be downloaded at: https://www.mdpi.com/article/10.3390/vaccines13030246/s1, Figure S1: Representative flow gating and quantification of isolated CD3+ CD8+ T cells for subsequent activation.
