# Supplementary material for: Type I Interferon Modulates the Function of Ly6C High-Expressing Naïve CD8+ T Cells to Promote an Antitumor Response
Source: Vaccines (Basel). 2025 Feb 27;13(3):246. doi: 10.3390/vaccines13030246 (PMC11945601; doi:10.3390/vaccines13030246)
Supplement: Supplementary file 1 [file vaccines-13-00246-s001.zip › vaccines-3467103-supplementary.pdf]

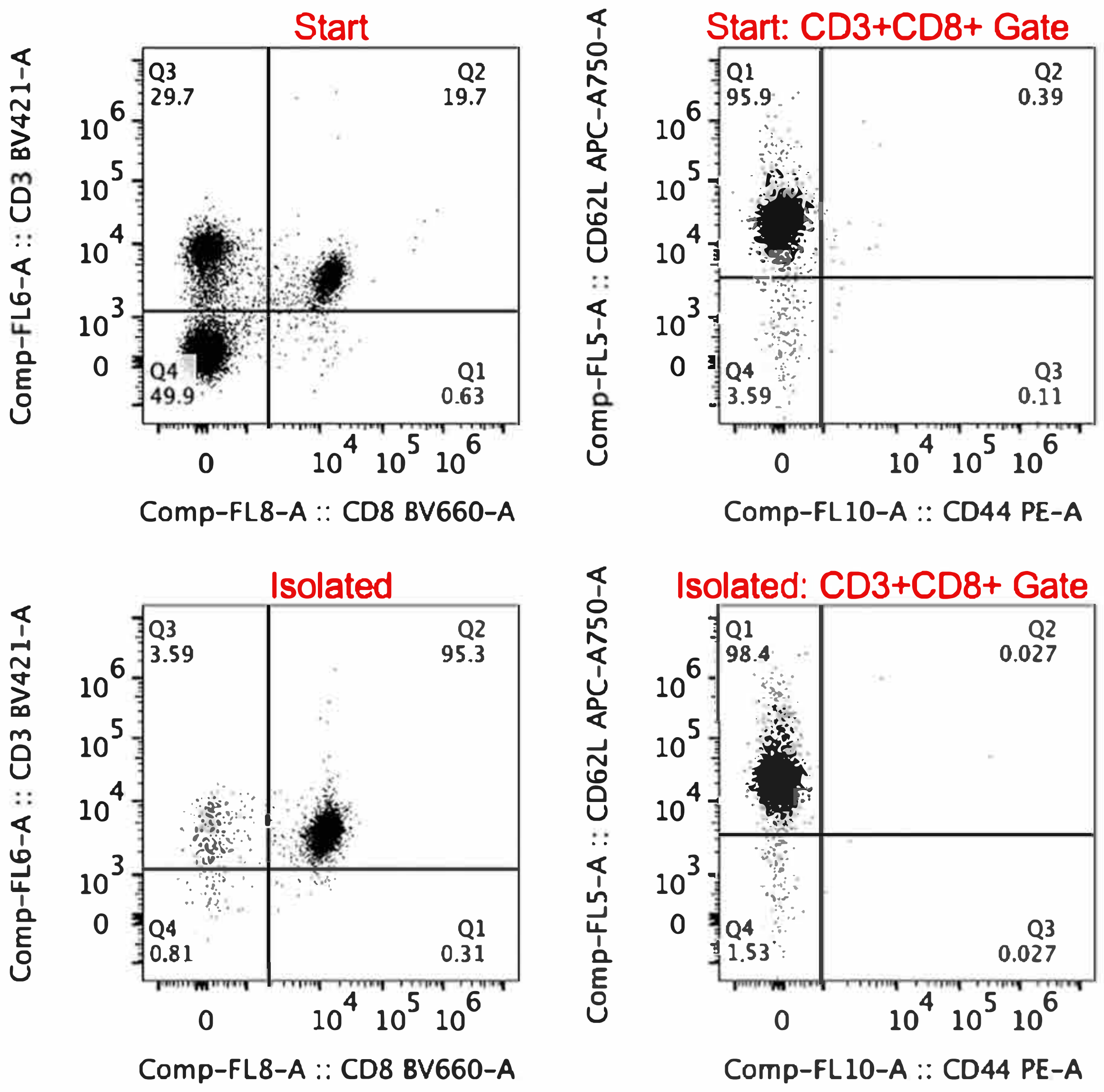


**Figure S1:** Representative flow gating and quantification of isolated CD3+ CD8+ T cells for subse-quent activation.
